# Roles of the subfornical organ and area postrema in arterial pressure increases induced by 48‐h water deprivation in normal rats

**DOI:** 10.1002/phy2.191

**Published:** 2014-01-06

**Authors:** John P. Collister, David B. Nahey, Michael D. Hendel, Virginia L. Brooks

**Affiliations:** 1 Department of Veterinary and Biomedical Sciences, College of Veterinary Medicine, University of Minnesota, St. Paul, 55108, Minnesota; 2 Department of Physiology & Pharmacology, Oregon Health and Science University Portland, Oregon, 97239

**Keywords:** Area postrema, blood pressure, heart rate, subfornical organ, water deprivation

## Abstract

In rats, water deprivation (WD) increases arterial blood pressure (BP) in part due to actions of elevated osmolality in the brain to increase vasopressin levels and sympathetic activity. However, the osmoreceptors that mediate this response have not been identified. To test the hypothesis that osmoregulatory circumventricular organs are involved, BP and heart rate (HR) were continuously recorded telemetrically during 48 h of WD in normal rats with lesions (x) or sham lesions (sham) of the subfornical organ (SFO) or area postrema (AP). Although WD increased BP in SFOx and SFOsham rats, no significant difference in the hypertensive response was observed between groups. HR decreased transiently but similarly in SFOx and SFOsham rats during the first 24 h of WD. When water was reintroduced, BP and HR decreased rapidly and similarly in both groups. BP (during lights off) and HR were both lower in APx rats before WD compared to APsham. WD increased BP less in APx rats, and the transient bradycardia was eliminated. Upon reintroduction of drinking water, smaller falls in both BP and HR were observed in APx rats compared to APsham rats. WD increased plasma osmolality and vasopressin levels similarly in APx and APsham rats, and acute blockade of systemic V1 vasopressin receptors elicited similar depressor responses, suggesting that the attenuated BP response is not due to smaller increases in vasopressin or osmolality. In conclusion, the AP, but not the SFO, is required for the maximal hypertensive effect induced by WD in rats.

## Abstract (precis)

The results describe the roles of the area postrema and subfornical organ in the hypertensive response to 48‐h water deprivation.

## Introduction

Water deprivation (WD) is associated not only with decreases in total body water but also in sodium secondary to a dehydration‐induced natriuresis (McKenna and Haines 1981; McKinley et al. 1983; Thrasher et al. 1984. Yet, despite the resulting significant decrease in extracellular fluid volume, arterial pressure rises (Gardiner and Bennett 1985; Blair et al. 1997; Scrogin et al. 2002; Veitenheimer and Osborn 2011, 2013; Veitenheimer et al. 2012. Previous work suggests that increased osmolality contributes to the hypertensive response via elevations in vasopressin and sympathetic nerve activity (Gardiner and Bennett 1985; Brooks et al. 2005a. However, the specific brain sites that house the osmoreceptors that activate these hypertensive pathways are unknown.

Central osmolality is sensed largely by specific neurons within the circumventricular organs (CVOs), a discrete set of brain sites that lack the blood–brain barrier and are accessible to both the circulating blood as well as cerebrospinal fluid (Gross and Weindl 1987. One particular CVO, the organum vasculosum of the lamina terminalis (OVLT), has been implicated in the increases in vasopressin and renal sympathetic nerve activity evoked by acute hyperosmolality (for reviews, see McKinley et al. (2004); Stocker et al. (2008)). OVLT neurons are directly activated by increases in osmolality (Ciura et al. 2011, and OVLT lesions attenuated renal sympathoexcitation induced by acute increases in central osmolality (Shi et al. 2007. Interestingly, OVLT lesions did not affect the rise in blood pressure following increased central osmolality suggesting that even though it may mediate some degree of sympathoexcitation during hyperosmotic states, it does not mediate the pressor response to increased osmolality (Shi et al. 2007. Nonetheless, previous work suggests that other CVOs, the area postrema (AP) and subfornical organ (SFO), are also likely involved. Notably, the AP and SFO have known anatomical connections to sympathetic regulatory centers, such as the paraventricular nucleus of the hypothalamus (PVN) and rostral ventrolateral medulla (RVLM) (Shapiro and Miselis 1985; Wilson and Bonham 1994; Anderson et al. 2001, and have been shown to mediate in part the increases in vasopressin in response to cellular dehydration (Huang et al. 2000; McKinley et al. 2004. In addition, another pressor hormone increased by WD, angiotensin II, acts via both the AP and SFO to acutely and chronically elevate blood pressure and sympathetic activity (Mangiapane and Simpson 1980; Casto and Phillips 1984; Fink et al. 1987a; Hendel and Collister 2005.

Therefore, the purpose of the present experiments was to test the following hypothesis: the pressor response induced by 48 h of WD in rats requires an intact AP or SFO. To test this hypothesis, it was determined if the increases in arterial pressure were reduced in rats with lesions of the AP or SFO.

## Materials and Methods

### Surgical methods

All experiments were conducted at the University of Minnesota and approved by the Institutional Animal Care and Use Committee. Adult male Sprague‐Dawley rats (275–325 g) were randomly selected for either lesion of the area postrema (APx; *n* = 7), subfornical organ (SFOx; *n* = 6), or respective sham (APsham; *n* = 7 or SFOsham; *n* = 6) operation. For all surgeries, rats were preanesthetized with pentobarbital (32.5 mg/kg, IP) and atropine (0.2 mg/kg, IP), and surgical anesthesia was achieved with a second injection containing a cocktail of anesthetic agents (acetylpromazine, 0.2 mg/kg; butorphanol tartrate, 0.2 mg/kg; ketamine, 25 mg/kg, IM). Rats received an intramuscular antibiotic injection of 2.5 mg gentamycin and a subcutaneous injection of 0.075 mg butorphanol tartrate for analgesic purposes postoperatively.

For APx surgeries, rats were placed in a stereotaxic device with the neck flexed. The AP was visualized through an incision between the occipital crest and the first vertebrae and was removed by suction using a blunt 25‐gauge needle attached to a vacuum line. Sham operations were identical except for the attached vacuum line. For SFOx surgeries, rats were placed in a stereotaxic device and the head was leveled. A 3 mm hole was drilled in the top of the skull just caudal to bregma, and a Teflon‐insulated monopolar tungsten electrode was lowered into four predetermined coordinates (Hendel and Collister 2005 within the SFO, through which a 1 mA current was passed for 7 sec. For sham operations, the electrode was not lowered as deeply into the brain and no current was passed.

Rats were allowed 1–2 weeks of recovery before implantation of radiotelemetric pressure transducers (model no. TA11PA‐C40, Data Sciences International, St. Paul, MN) and femoral catheters as described previously (Hendel and Collister 2005 for continuous blood pressure and heart rate monitoring and blood sampling, respectively. Briefly, a midline abdominal incision was made and the descending aorta was exposed. The aorta was clamped and the catheter of the transducer was introduced distal to the clamp and glued in place. The aortic clamp was released, and the transmitter unit was attached to the abdominal wall with 3–0 surgical suture during closure of the abdominal cavity. Next, a small ventral incision was made in the left leg and the femoral vein exposed. The vein was tied off and the catheter introduced approximately 9 mm into the vein and tied in place. The catheter was then tunneled subcutaneously to an exit location between the scapulae and passed through a flexible spring connected to a single‐channel hydraulic swivel. After transmitter and catheter implantation, rats were given another week of recovery. During all recovery periods rats were given standard rat chow and distilled water ad libitum.

### Experimental procedure

Rats were maintained on a 12‐h light–dark schedule and mean arterial pressure (MAP) and heart rate (HR) were recorded via telemetry (500 Hz for 10 s each minute) continuously throughout the experiment. A control period of 2 days, during which rats were allowed standard rat chow and distilled water ad libitum, preceded the experimental phase. On the first experimental day, water was removed from the cages of all animals 4 h before the lights turned on. Water was withheld for 48 h, during which rats continued to have access to standard rat chow ad libitum. After the 48‐h WD, again at 4 h before lights on, water was reintroduced to the rats. The bottle was touched to the mouth of each rat upon reintroduction to ensure that each rat began drinking immediately. Water intake was measured for each rat at 1‐h intervals for the next 4 h. For vasopressin and osmolality measurements (APx and APsham rats), blood was drawn at approximately 10 min before water was removed and again 10 min before water was returned. In some rats (APx *n* = 3, APsham *n* = 3), a V1 vasopressin antagonist (Manning compound, [*β*‐Mercapto‐*β*,*β*‐cyclopenta‐methylenepropionyl^1^,O‐Me‐Tyr^2^, Arg^8^]‐vasopressin; MP Biomedicals, Santa Ana, CA) was injected iv (10 *μ*g/kg) immediately after blood was drawn at the end of the WD period, but before water was reintroduced. For all experiments, blood pressure and heart rate were measured for a 2‐day recovery period after reintroduction of water.

### Data collection

The MAP and HR data collected by telemetry were largely averaged in 12‐h blocks, representing the lights on and lights off periods. These 12‐h averages were collected for the lights on period the day before WD, during the first 24 h of WD, the 12‐h lights on period of the second day of WD, and the day following WD (recovery). During the lights off period just before WD and the lights off period of the second day of WD, only 8 h were averaged, up until water was removed or returned, 4 h before lights on. Instead, the data collected for 4 h after returning water were averaged in 1‐h blocks.

To analyze vasopressin (APx *n* = 5, APsham *n* = 2) and plasma osmolality (APx *n* = 3, APsham *n* = 3) responses in some rats, 0.5‐mL blood was drawn through the catheter of each rat into a chilled syringe containing EDTA. Blood was transferred to chilled microcentrifuge tubes, then centrifuged and separated, with the plasma being drawn off. Osmolality was measured with a vapor pressure osmometer (model 5500, Wescor, Logan UT), and plasma was stored at −80°C for later assay of vasopressin at the Core Lab of the Medical College of Wisconsin as described previously (Carlson et al. 1997. An equal volume of saline was injected through the catheter after the blood draw to minimize volumetric effects.

To determine if APx reduced the contribution of circulating vasopressin to MAP support, the average of data collected 15 min following V1 antagonist injection was compared to preinjection values averaged for the 10‐min period prior.

### Histological verification of lesions

**Figure 1.**
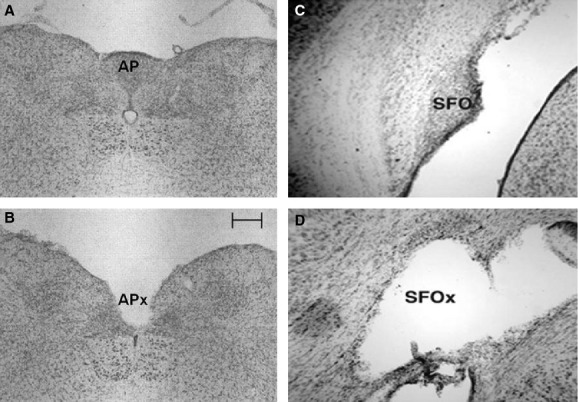
Photomicrographs of 40‐*μ*m coronal sections of the rat brain with an intact area postrema (A) and after lesion of the area postrema (B) (left panels), and midsagittal sections of the rat brain with an intact subfornical organ (C) and after lesion of the subfornical organ (D) (right panels). Scale bar = 500 *μ*m.

On completion of the protocol, all rats were anesthetized as described above and perfused intracardially with 140 mL of heparinized saline (20 U/mL heparin in 0.9% saline), followed by 450 mL of 4% paraformaldehyde in phosphate buffer saline (PBS). Whole brains were dissected and soaked in 4% paraformaldehyde for 2 days. The brains were then transferred to a 30% sucrose solution for a minimum of 2 days. For SFOx and SFOsham rats, frozen serial sagittal sections (50 *μ*m) were made at the lateral edge of the third ventricle and mounted on treated slides. For APx and APsham rats, coronal serial sections (50 *μ*m) were sliced and similarly mounted for inspection. Slides were stained for Nissl substance with cresyl violet and examined using light microscopy for confirmation of an intact (sham) or lesioned CVO. All APx rats included in the study were confirmed to have undergone complete AP ablation with minimal damage to the surrounding tissue. While our lesions were centered on the AP, in order to completely lesion the AP, it should be noted that invariably some fibers or inherent connections with the medial nucleus tractus solitarius (NTS) were damaged as well. All SFOx rats included in the final analysis of the data were confirmed to have ≥ 80% of the SFO ablated by the lesion, as well as complete removal of the rostroventral portion including efferent fibers of the SFO. Figure 1 illustrates representative examples of lesioned and sham rats.

### Statistical analysis

Within the lights on or lights off periods, between‐group differences in both the absolute and the changes in MAP and HR were assessed using two‐way ANOVA for repeated measures [factors are group (lesion, sham) and time] and the post hoc Tukey–Kramer test. Between‐group differences in the decreases in MAP and HR immediately following reintroduction of water were assessed using *t*‐tests to determine if the changes in MAP and HR before and 4 h after return of water differed between groups. A two‐way ANOVA for repeated measures was also used to determine if there was a between‐group difference in the effect of WD on plasma vasopressin levels. Finally, a *t*‐test was used to detect any difference in the MAP response to V1 antagonism in APx and APsham rats. All results are reported as mean ± standard error and a critical value of *P* < 0.05 (two‐sided) was considered statistically significant for all tests.

## Results

### Effects of WD on MAP and HR

**Figure 2.**
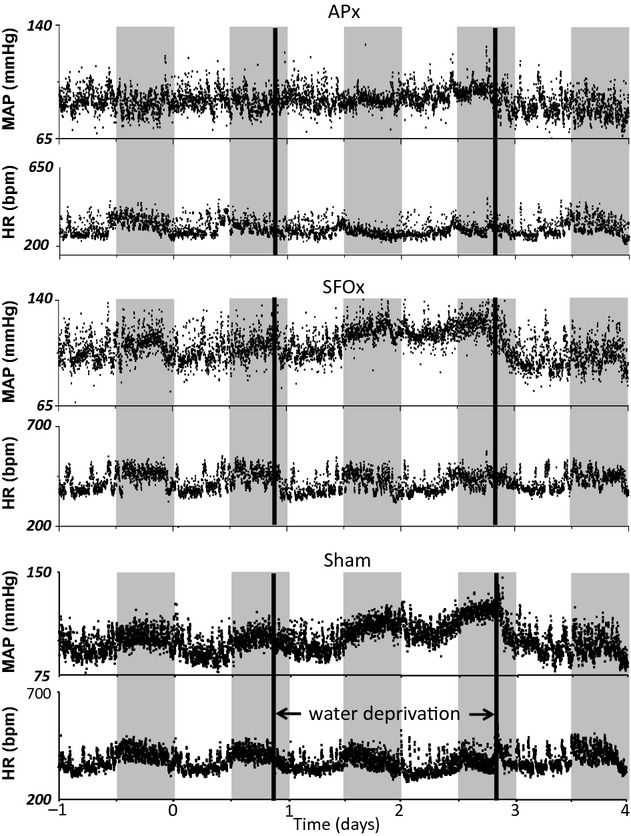
Representative tracings from an APx rat (top panel), a SFOx rat (middle panel), and a sham‐operated rat (bottom panel) showing mean arterial pressure (MAP) and heart rate (HR) before, during, and after a period of 48‐h water deprivation. Gray stripes indicate the lights off segment of the day–night cycle.

**Figure 3.**
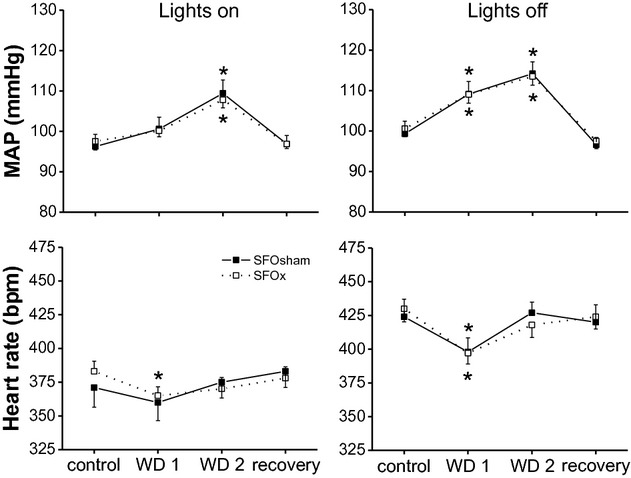
Mean arterial pressure (MAP) and heart rate responses to 48‐h water deprivation in rats with an intact subfornical organ (SFOsham, filled squares) and with lesion of the SFO (SFOx, open squares) during the lights on (left) and lights off (right) segments of the day–night cycle. (*P < 0.05 compared to baseline).

**Figure 4.**
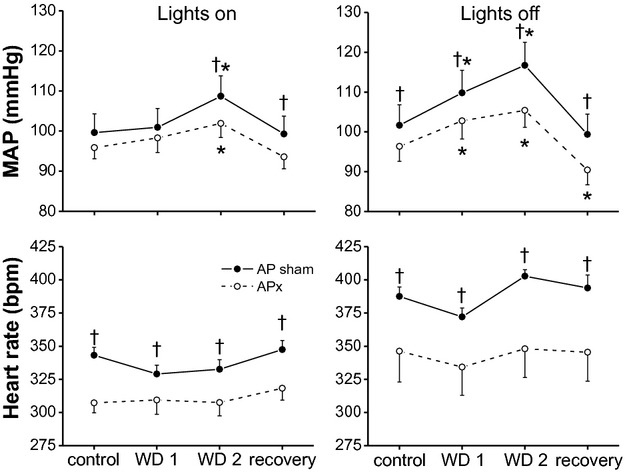
Mean arterial pressure (MAP) and heart rate (HR) responses to 48‐h water deprivation in rats with an intact area postrema (APsham, closed circles) and with lesion of the AP (APx, open circles) during the lights on (left) and lights off (right) segments of the day–night cycle. While WD did not decrease HR compared to baseline, HR was suppressed (*P* < 0.05) in the APsham rats, but not APx rats, after 24 h of WD (lights on and lights off) compared to the recovery period. (*P < 0.05 compared to baseline; ^†^*P* < 0.05 between groups).

As illustrated by a continuous representative tracing (Fig. 2, bottom panel) and the combined results (Figs. 3, 4 from control sham‐lesioned rats, MAP rose gradually during WD, to reach significantly elevated levels during the dark phase 24 h after removing water. HR decreased transiently, but returned to baseline by the end of the 2‐day WD period.

### Effects of SFOx

Figure 3 illustrates the MAP and HR responses to 48‐h WD in SFOsham and SFOx rats. A typical experimental tracing from an individual SFOx rat is shown in Figure 2 (middle panel). Baseline MAP and the MAP responses to WD did not differ between the lesioned and sham‐lesioned groups. Baseline HR also did not differ between groups and fell similarly and transiently during the light and dark phases on the first day of WD. MAP and HR both decreased (*P* < 0.05) rapidly upon reintroduction of water (Fig. 2; however, the falls in MAP (−18 ±2 mmHg, SFOsham; −19 ± 2 mmHg, SFOx; *P* > 0.10) and HR (−104 ± 5 bpm, SFOsham; −86 ± 11 bpm, SFOx; *P* > 0.10) 4 h after water was returned were similar between groups.

### Effects of APx

Mean arterial pressure and HR responses to WD in APx and APsham rats are shown in Figure 4. Baseline MAP was lower in APx rats during the dark phase. Both groups of rats exhibited significant increases in MAP after 48 h WD, but the increase was attenuated in APx rats during the lights off phase (APx: 9.0 ± 1.5 mmHg; APsham: 15.1 ± 1.5 mmHg; *P* < 0.05). Baseline HR was lower in APx rats compared to APshams throughout the light–dark cycle. WD decreased HR during the first 24 h (compared to recovery but not baseline) in the APsham but not APx rats. Nevertheless, significant between‐group differences in the changes in HR during and immediately following WD were not observed. Following reintroduction of water, both groups demonstrated significant decreases in MAP and HR (*P* < 0.05); however, the decreases in MAP (−20 ± 1 mmHg, sham; −16 ± 1 mmHg, APx) and HR (−93 ± 11 bpm, sham; −56 ± 12 bpm, APx) were attenuated (*P* < 0.05) in APx rats compared to APsham rats at 4 h after the return of water.

### Does APx alter the vasopressin or hyperosmolar response to WD?

Water deprivation robustly increased (*P* < 0.05) plasma vasopressin levels in APx rats from 4.4 ± 0.9 to 16.2 ± 1.8 pg/mL and in APsham animals from 3.6 ± 0.1 to 18.2 ± 1.9 pg/mL. This rise in vasopressin was not different between groups. In addition, while iv injection of the V1 antagonist lowered MAP in both groups (*P* < 0.05), the depressor responses were not different (−3.7 ± 1.4 mmHg, APsham, *n* = 3; −4.3 ± 1.0 mmHg, APx, *n* = 3). WD increased osmolality (*P* < 0.05) similarly in APx and APsham rats. In APx (*n* = 3) rats, it rose from 304 ± 2 mOsm/kg H_2_O before the 48‐h deprivation to 309 ± 2 mOsm/kg H_2_O, whereas in APsham rats (*n* = 3) osmolality increased from 300 ± 4 to 307 ± 1 mOsm/kg H_2_O before and after WD, respectively.

## Discussion

The primary purpose of this study was to test the hypothesis that the SFO and/or the AP mediate the rise in arterial pressure induced by WD, using conscious rats instrumented for telemetric recordings of arterial pressure and heart rate. We confirm using telemetry (Veitenheimer and Osborn 2011, 2013; Veitenheimer et al. 2012 that (1) WD elicits a gradual and progressive pressor response that is evident within 24 h and reaches a peak increment of ~15–20 mmHg after 48 h; (2) the rise in arterial pressure is accompanied by a transient bradycardia; and (3) both MAP and HR rapidly decrease when water is returned to the rats after 48 of WD. Our novel findings are that (1) SFO lesions do not reduce the pressor response; and (2) AP lesions attenuate the WD‐induced pressor response by 40% and abolish the transient bradycardia. Collectively, these data suggest that the AP, but not the SFO, contributes to the changes in MAP and HR induced by WD.

It is well established that, when water intake is prevented, obligatory water losses from the skin, lungs, and kidney result in decreased total body water, as evidenced by an increase in plasma osmolality. In addition, total body sodium is reduced, due to both decreased food intake and also increased urinary sodium excretion, with subsequent hypovolemia. Despite these reductions in water and sodium, at least in rats, arterial pressure rises (Gardiner and Bennett 1985; Blair et al. 1997; Scrogin et al. 2002; Veitenheimer and Osborn 2011, 2013; Veitenheimer et al. 2012. Three pressor pathways have been shown to contribute to this pressor response: angiotensin II, vasopressin, and the sympathetic nervous system (Gardiner and Bennett 1985; Scrogin et al. 1999, 2002. Decreases in blood volume and subsequent arterial and cardiopulmonary baroreflex activation underlie in part the activation of the renin–angiotensin system, vasopressin release (Blair et al. 1997; Gottlieb et al. 2006, and likely also increases in sympathetic nerve activity. However, arterial pressure “overshoots” these homeostatic mechanisms to rise above normal because of a central effect of elevated osmolality to drive the sympathetic nervous system and stimulate vasopressin (Scrogin et al. 1999; Brooks et al. 2005a; Gottlieb et al. 2006. Moreover, central synergistic interactions between osmolality and angiotensin II may lead to excessive sympathetic activation, which contributes to the hypertension that is produced (Gardiner and Bennett 1985; Brooks et al. 2005b; Veitenheimer et al. 2012. However, although considerable evidence implicates key roles for PVN (Stocker et al. 2004a; Stocker et al. 2005; Freeman and Brooks 2007 and the RVLM (Brooks et al. 2004a,b; Stocker et al. 2006, the brain neuronal circuitry involved in these integrated responses has not been completely mapped. In particular, the brain osmosensitive sites that trigger these pressor pathways have not been identified.

Several experimental approaches have provided key information implicating the CVOs that mediate the sensation of osmolality and angiotensin II during WD, in particular, quantification of Fos expression as an index of activated neurons and specific lesions of CVOs. Direct recordings of isolated SFO neurons reveal that these cells are stimulated by hypertonicity (Anderson et al. 2000. Moreover, Fos studies indicate that the SFO is activated, albeit modestly, following 48 h of WD (McKinley et al. 1994; Morien et al. 1999; Sly et al. 2001; De et al. 2002. Interestingly, a significant fraction of the activated neurons project to the supraoptic nucleus, thereby capable of increasing vasopressin secretion (McKinley et al. 1994, while another fraction projects indirectly to the kidney via the sympathetic nervous system (Sly et al. 2001. The SFO mediates the hypertension resulting from chronic infusion of low doses of angiotensin II, which acts in part via sympathoexcitation (Zimmerman et al. 2004; Collister and Hendel 2005, and WD can increase the expression of angiotensin II AT1 receptors in the SFO (Sanvitto et al. 1997. In sheep, SFO lesions markedly reduced the increase in vasopressin induced by acute increases in osmolality (McKinley et al. 2004; in contrast, SFO lesions were without effect in rats (Maliszewska‐Scislo et al. 2008. Therefore, significant indirect evidence supports a potential role for the SFO in the pressor response. However, the present results indicate that SFO lesions were largely ineffective. One interpretation of this result is that the SFO does not play a major role in the hypertensive response. This interpretation is supported by the relatively low induction of Fos in the SFO after WD compared to other brain regions such as the OVLT, median preoptic nucleus, SON, and PVN (McKinley et al. 1994; Morien et al. 1999; De et al. 2002, as well as the failure of SFO lesions to attenuate the vasopressin response to acute increases in osmolality (Maliszewska‐Scislo et al. 2008. An alternate interpretation is that the SFO does provide a significant contribution, but redundant mechanisms provided by the OVLT and AP can compensate when this site is eliminated.

Indirect evidence also implicates the AP in the pressor response induced by WD. WD elicits increased Fos expression in the AP (Gottlieb et al. 2006, and AP lesions attenuate hypertension secondary to angiotensin II infusion (Fink et al. 1987a as well as the increase in vasopressin triggered by acute systemic increases in osmolality (Huang et al. 2000. In this study, AP lesions abolished the bradycardia and markedly reduced the pressor response to WD. Therefore, we conclude that the AP is required for WD to reduce HR and to maximally increase arterial pressure in rats. Nevertheless, a significant component of the pressor response remained in APx rats, suggesting that another site, likely the OVLT, is also involved.

One possible explanation of the reduced pressor responses in WD APx rats is that the participation of vasopressin is reduced, as AP lesions have been shown to blunt the rise in vasopressin following acute hypertonicity (Huang et al. 2000. However, this possibility is unlikely for the following reasons. First, the contribution of vasopressin to arterial pressure maintenance during WD and following acute increases in osmolality are small (Kawano et al. 1991; Scrogin et al. 1999. Second, the ability of AP lesions to reduce the vasopressin response to iv hypertonic saline administration was not evident until osmolality exceeded levels normally induced by WD (Huang et al. 2000. Finally, we found that the rise in vasopressin produced in WD AP lesioned rats is quite robust and not different from sham‐lesioned animals. Moreover, the depressor responses to acute V1 vasopressin receptor blocker were not different in APsham and APx rats. Therefore, while our low sample sizes may have precluded the detection of a small vasopressin contribution, we conclude that reductions in the levels or actions of vasopressin are not a major factor in the ability of APx to attenuate the WD pressor response.

As AP lesions can impair food intake (Hyde and Miselis 1983; Johnson and Gross 1993; Collister and Hendel 2003 and the rise in osmolality induced by WD depends on food intake, the smaller pressor response in AP‐lesioned rats could have resulted from a smaller degree of hypertonicity. However, AP‐lesioned rats have normal basal plasma osmolalities and exhibit normal increases in osmolality in response to acute elevations in osmolality (Huang et al. 2000 and WD. Indeed, in this study, baseline osmolality was not different in APx and APsham rats, and furthermore increased to similar levels after 48 h WD.

Another possibility is that the lesion attenuated the sympathoexcitation known to be induced by increases in osmolality during WD (Brooks et al. 1997; Scrogin et al. 1999; Veitenheimer et al. 2012. In support of this hypothesis, AP lesions have been shown to reduce the pressor and tachycardic responses following intracisternal hypertonic saline infusion (Kawano et al. 1991. Moreover, the AP is known to project directly or indirectly (via the NTS) to the PVN and RVLM (Shapiro and Miselis 1985; Blessing et al. 1987; Wilson and Bonham 1994, two brain regions that contribute to arterial pressure maintenance and increases in sympathetic nerve activity during WD (Brooks et al. 2004a,b; Stocker et al. 2004a,b; Stocker et al. 2005; Stocker et al. 2006;; Freeman and Brooks 2007. Indeed, while our lesions were focused on the AP, it should be noted that some fibers or medial NTS dendritic processes projecting to the AP were likely damaged as well. Lastly and interestingly, the AP also projects to the nucleus ambiguus (Shapiro and Miselis 1985, which may explain its involvement in the decreases in HR observed in WD rats. Nevertheless, further experiments are required to directly test this hypothesis.

### Perspectives

Like WD, increased dietary salt increases plasma osmolality (for reviews, see Brooks et al. (2005b); De Wardener et al. (2004); He et al. (2005)). Normally, the increases in osmolality are small and do not trigger the same pressor pathways as WD (e.g., sympathoexcitation and vasopressin), as a simultaneously expanded blood volume suppresses the renin–angiotensin–aldosterone system. Indeed, if anything sympathetic activity may actually decrease (Brooks and Osborn 1995; Brooks et al. 2005b. However, in humans and experimental models of salt‐sensitive hypertension, increased dietary salt activates the sympathetic nervous system (Carlson et al. 2001; Leenen et al. 2002; Brooks et al. 2005b. One mechanistic explanation of this adverse pressor response is that concurrent inappropriate elevations of hormones such as angiotensin amplify the sympathoexcitatory actions of the small increases in osmolality (Brooks et al. 2001, 2005b; Osborn et al. 2007. Thus, the neuroendocrine‐cardiovascular picture presented by salt‐sensitive hypertension is similar to WD. The present results reveal that the SFO is not required for the WD‐induced pressor response, yet the AP makes a major contribution. Given the parallels between WD and salt‐sensitive hypertension, although the AP does not appear to modulate BP during changes in dietary salt in the normal rat (35), we speculate that the AP may play a greater role than the SFO in the genesis of sympathoexcitation in salt‐sensitive individuals as well. In support of this hypothesis, previous studies have shown that lesions of the AP (Fink et al. 1987b, but not the SFO (Osborn et al. 2006, reduce arterial pressure in DOCA‐salt rats. Nevertheless, further experimental work is required to test this hypothesis, as well as the role of other key osmoregulatory CVOs, such as the OVLT.

## Conflict of Interest

None declared.
